# The tale of capturing Norrin

**DOI:** 10.7554/eLife.98933

**Published:** 2024-05-30

**Authors:** Hsin-Yi Henry Ho

**Affiliations:** 1 Department of Cell Biology and Human Anatomy, University of California, Davis Davis United States

**Keywords:** signaling, Norrie disease, Wnt/β-catenin pathway, Norrin, Tspan12, None

## Abstract

Detailed binding experiments reveal new insights into the Norrin/Wnt signaling pathway that helps to control vascularization in the retina.

**Related research article** Bruguera ES, Mahoney JP, Weis WI. 2024. The co-receptor Tspan12 directly captures Norrin to promote ligand-specific β-catenin signaling. *eLife* **13**:RP96743. doi: 10.7554/eLife.96743.

Many ocular disorders can be traced to blood vessels forming improperly in the eye. In two related genetic conditions known as Norrie disease and familial exudative vitreoretinopathy (FEVR), for example, incomplete development of the retinal vasculature leads to aberrant formation of new blood vessels (known as neovascularization), abnormal tissue growths and the wrinkling of retinal layers (Figure 1A). This often results in retinal detachment and ultimately blindness. Understanding the genetic and molecular processes that contribute to these conditions can help to uncover new treatments, while also providing valuable insights into developmental processes.

**Figure 1.**
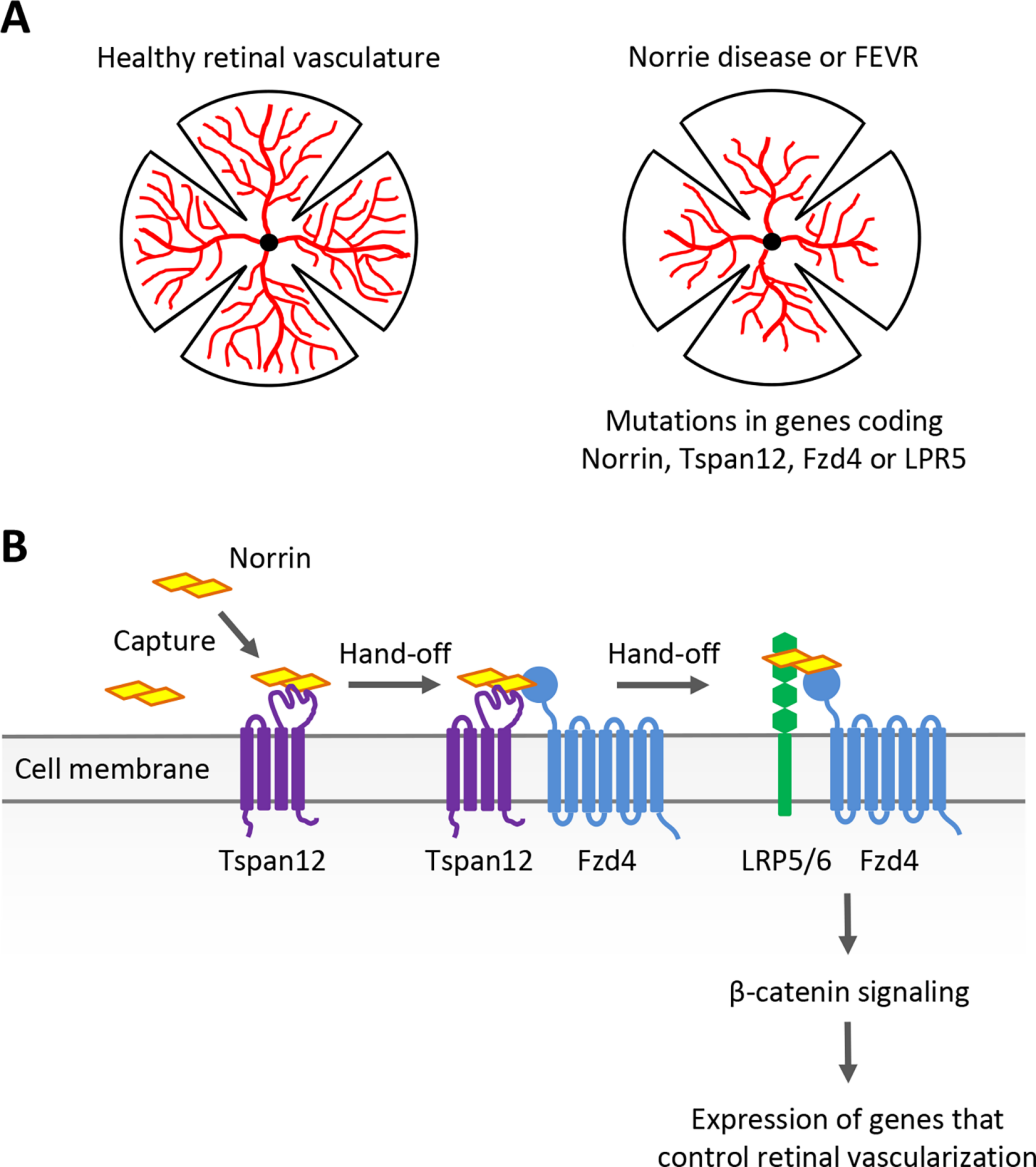
Examining the role of Norrin, Tspan12, Fzd4 and LRP5/6 in retinal blood vessel development. (**A**) Norrie disease and familial exudative vitreoretinopathy (FEVR), which are linked to mutations in the genes encoding Norrin, Tspan12, Fzd4 or LRP5, are associated with defects in the vascularization (red) of the retina. (**B**) The signaling protein Norrin (yellow) and transmembrane receptors Tspan12 (purple), Fzd4 (blue) and LRP5/6 (green) function in a common signaling pathway to activate a genetic program that controls retinal vascularization. Bruguera et al. propose a molecular model in which Tspan12 captures Norrin (left) and hands it off to Fzd4 and the Fzd4-LRP5/6 receptor complex (right) to activate downstream signaling.

Norrie disease and FEVR have been linked to mutations in the gene coding for the secreted signaling protein Norrin. Changes to the genes for the Fzd4, LRP5 and Tspan12 transmembrane proteins are also known to cause FEVR (Gilmour, 2015). Great progress has been made over the past two decades in establishing how these molecular actors contribute to blood vessel development in the retina, showing in particular that they are involved in the Wnt/β-catenin signaling pathway (Xu et al., 2004; Ye et al., 2009, Junge et al., 2009; Ke et al., 2013; Chang et al., 2015).

Classically, secreted Wnt proteins bind to transmembrane receptors of the Frizzled (Fzd) family – including Fzd4 – with the help of the co-receptor LRP5 or the related protein LRP6. This triggers a series of molecular events which result in β-catenin activating developmental genes that control how cells divide, differentiate and migrate. Norrin does not share any structural similarities with Wnt proteins, yet it can mimic their function by engaging Fzd4 and LRP5 to trigger the same downstream β-catenin pathway during retinal development (Xu et al., 2004; Figure 1B). This process requires Norrin to bind to the co-receptor Tspan12, even though this protein is not normally required for Wnt signaling (Junge et al., 2009). Exactly why this is the case and how it occurs has remained unclear. Addressing these questions requires precise measurement of the interactions between Tspan12, Norrin, Fzd4 and other receptors. Now, in eLife, Elise Bruguera alongside colleagues Jacob Mahoney and William Weis at Stanford University School of Medicine report having bypassed certain methodological limitations to explore this variation of the Wnt signaling pathway in detail (Bruguera et al., 2024).

Receptors tend to aggregate and lose their activity once extracted from the membrane, making them difficult to study using conventional approaches. Instead, the team purified and embedded Tspan12 and other Norrin co-receptors into nanodiscs; these well-characterized synthetic scaffolds of lipid bilayers resemble the natural environment of the receptors while also allowing precise measurements of their binding affinity (Bruguera et al., 2022).

Strikingly, the experiments showed that Tspan12 binds Norrin with exceedingly high affinity, even in the absence of other co-receptors such as Fzd4. Bruguera et al. used the powerful artificial intelligence program AlphaFold to predict potential binding sites between Norrin and Tspan12. Of the four sites returned by these analyses, three were confirmed via experiments examining how Norrin and Tspan12 could bind after the charge or shape of amino acids at these domains had been altered. Importantly, a mutation associated with Norrie disease is found at one of these sites, strengthening the validity of these findings.

Next, Bruguera et al. addressed how Tspan12 could help Norrin trigger Fzd4- and LRP5/6-mediated β-catenin signaling. Several potential mechanisms were considered and systematically tested, with AlphaFold predictions being used to guide the required experiments. Having shown that Norrin can bind both Tspan12 and Fzd4 simultaneously, the team investigated whether Tspan12 could allow Fzd4 to have a higher affinity for Norrin (by forming a complex with the receptor), or to better bind downstream signaling components (by altering its conformation). However, the analyses showed that this was not the case. In turn, examining if Tspan12 could act by increasing Norrin’s ability to bind LRP5/6 highlighted that, in fact, high Tspan12 levels inhibited Norrin-LRP5/6 associations.

Still, experiments with nanodiscs showed that Fzd4 could bind Norrin more efficiently when Tspan12 was present at the membrane – especially when Norrin was in miniscule concentrations. Likewise, cells expressing both Tspan12 and Fzd4 were better at detecting low levels of Norrin and initiating β-catenin signaling than cells expressing Fzd4 alone. Such findings are consistent with Tspan12 facilitating the detection of Norrin by providing extra binding sites for it on the cell surface.

Taken together, these results led Bruguera et al. to propose that Tspan12 captures Norrin – particularly when present in low quantities – and therefore increases its local concentration at the membrane. As Tspan12 is often located near Fzd4, it can then pass Norrin to Fzd4, which proceeds to form a Norrin-Fzd4-LRP5/6 complex that triggers downstream β-catenin signaling (Figure 1B). This clever strategy, which relies on subtle differences in binding affinity and compatibility between Norrin and its co-receptors, allows cells to sense even small amounts of Norrin, and potentially gradients. Such sensitivity is likely essential; the location and levels of Norrin production are carefully regulated during retinal development, and cells need to be able to accurately respond to this intricate expression pattern (Ye et al., 2009).

More generally, this variation to traditional Wnt signaling represents a prime example of how adaptations can be built into existing biological frameworks to create functional diversity and specificity. Similar mechanisms likely exist in other pathways to facilitate how signals are sensed. In the future, it will be interesting to investigate how Norrin is passed from Tspan12 to the Tspan12-Fzd4 and Fzd4-LRP5/6 complexes, and whether additional ‘exchange-promoting’ factor(s) are required.

Finally, the work by Bruguera et al. has important clinical implications. Current therapies for neovascularization rely on drugs that block blood vessel generation altogether, causing unwanted side effects when administered systemically. As a result, these inhibitors must be delivered locally through repeated ocular injections. Improper neovascularization being restricted to the retina in Norrie disease and FEVR suggests that this process is controlled locally by Norrin and Tspan12; approaches that target these proteins should therefore act more selectively, even if administered systemically. The biochemical and structural insights from the study by Bruguera et al. will undoubtedly advance the development of such therapies.
